# Development and validation of the Pediatrics Functional Living Index—Emesis scale

**DOI:** 10.3389/fonc.2025.1573996

**Published:** 2025-06-24

**Authors:** Lili Han, Jun Deng, Yuyun Yang, Wanqi Yu, Wenxia Zhang, Jieru Lin, Feifei Zuo, Jing Yu, Ruiqing Cai, Meiling Liu

**Affiliations:** ^1^ Department of Head and Neck Surgery, Sun Yat-sen University Cancer Center, State Key Laboratory of Oncology in South China, Guangdong Provincial Clinical Research Center for Cancer, Guangzhou, China; ^2^ Department of Pediatric Oncology, Sun Yat-sen University Cancer Center, State Key Laboratory of Oncology in South China, Guangdong Provincial Clinical Research Center for Cancer, Guangzhou, China

**Keywords:** pediatrics, cancer, nausea, vomiting, quality of life

## Abstract

**Background and objective:**

Assessing the impact of chemotherapy-induced nausea and vomiting (CINV) on the quality of life (QoL) of cancer patients is critical. However, there is a dearth of specialized assessment tools designed specifically for pediatric cancer patients. The aim of this study was to develop and validate the Pediatrics Functional Living Index-Emesis (PFLIE) as a patient-reported outcome measure (PROM) to assess the impact of CINV on QoL in pediatric patients. This study was approved by the Institutional Review Board of Sun Yat-sen University Cancer Center (Approval No. B2021-113-01) and was conducted in accordance with the Declaration of Helsinki.​

**Materials and methods:**

The reliability, content validity, structural validity, and concurrent validity of the PFLIE were assessed through two rounds of Delphi expert consultation and a questionnaire survey of 90 pediatric cancer patients receiving chemotherapy at a tertiary care hospital cancer center in China.

**Results:**

The PFLIE consists of two domains: nausea (10 items) and vomiting (10 items). The content validity index (CVI) for both the nausea and vomiting domains was 0.933. The Cronbach’s alpha coefficients for the total scale, nausea domain, and vomiting domain were 0.964, 0.928, and 0.943, respectively. Item-domain correlations were stronger for the PFLIE (r = 0.678-0.882) across domains compared to across-domain correlations (r = 0.493-0.780), suggesting that the PFLIE has acceptable construct validity. In addition, the PFLIE demonstrated acceptable concurrent validity.

**Conclusions:**

The validity and reliability of the Chinese version of the PFLIE are reliable and valid. The tool can help healthcare providers effectively identify and manage CINV symptoms, thereby improving the QoL of pediatric cancer patients. In low- and middle-income countries (LMICs) with limited resources, PFLIE can be used to improve the management of CINV and to ensure that pediatric cancer patients receive adequate care despite inadequate healthcare infrastructures. The tool can be used to improve the management of CINV and to ensure that pediatric cancer patients receive adequate care despite inadequate healthcare infrastructures.​

## 1 Introduction

CINV is a common and distressing side effect of chemotherapy in patients undergoing cancer treatment, including children (1). It can occur either acutely (0–24 hours) or in a delayed manner (24–120 hours) after chemotherapy (2). CINV is associated with a significant decrease in QoL and is perceived by patients as a major side effect of treatment (3). For pediatric patients, poorly controlled CINV can lead to nutritional deficiencies, weight loss, fatigue, increased risk of infection, and disruption of childhood activities, such as schooling (4). Consequently, international pediatric oncology organizations have issued recommendations for the prophylaxis of CINV in children (5–7). Despite the existence of well-established guidelines, adherence to antiemetic guidelines in the pediatric population is only approximately 20%-60%, which is lower than that observed in adults (8). Pediatric cancer patients represent a particularly unique and vulnerable group at risk for delayed detection and management of CINV. This risk predominantly arises from substantial variations in cognitive, linguistic, and both gross and fine motor skills, which are heavily influenced by the patient’s age, developmental phase, and the extent of their condition (9). One study suggested that self-reported CINV is reliable and accurate in pediatric patients starting at the age of 5 years (10). As noted earlier, reporting children’s own experiences with CINV in their daily lives can provide parents, caregivers, health care professionals, and policymakers with valuable insights into the specific needs of these children and inform the provision of more comprehensive and targeted services (11). However, in pediatric cancer treatment, research has often focused on the frequency and management of nausea and vomiting symptoms, while studies examining the impact of these self-reported symptoms on the daily life of pediatric patients remain limited.

The Functional Living Index-Emesis (FLIE) instrument, developed and validated by Lindley et al., is a PROM used to evaluate the impact of CINV on daily functioning and QoL (12). In its original form, the FLIE was designed with a three-day recall period. Subsequently, Martin et al. in 2003 verified that a 5-day recall version of the FLIE had sufficient measurement properties for assessing the impact of CINV on patients’ daily lives throughout both the acute and delayed phases following chemotherapy initiation (13). The FLIE consists of 18 items categorized into two domains: Nausea (items 1-9) and Vomiting (items 10-18). It focuses on how nausea and vomiting affect physical activity, social and emotional functioning, and the enjoyment of meals (12). Patients complete the FLIE questionnaire on the sixth day of their first chemotherapy cycle, evaluating the impact of CINV on their daily functioning over the 120-hour period following chemotherapy administration. In each domain, the first item requires patients to rate the intensity of nausea (or vomiting) experienced in the past 5 days. The subsequent eight items assess how nausea (or vomiting) affects various aspects of a patient’s daily life, including their ability to perform normal recreational and leisure activities, household tasks, enjoyment of meals and liquids, willingness to spend time with family and friends, performance of daily functions, and the extent to which the side effect has caused personal hardship and hardship for others. Each item is rated using a 100-mm visual analog scale (VAS) with anchors corresponding to “none/not at all” and “a great deal,” divided into six equal categories (1–7 points). Items within each domain are weighted equally and summed to create domain scores, which are then combined to generate a total score. Domain scores range from 9 to 63, with higher scores indicating less impairment in daily life due to nausea or vomiting. Overall combined domain scores greater than 108 points (i.e., scores greater than 54 points for each domain) have been shown to indicate no significant impact on daily life (12, 13).

The FLIE instrument has been translated into more than 20 languages (14), including Chinese (15), and has been widely used by researchers worldwide to measure the impact of CINV on daily living in adult cancer patients. However, to our knowledge, this instrument has not yet been applied to pediatric cancer patients. Therefore, based on the contents of the FLIE scale, combined with a literature review and clinical experience, this study employed the Delphi method (16) to develop the PFLIE scale and to test its reliability and validity in the Chinese context.

## 2 Materials and methods

### 2.1 Development and validation process

**Figure 1:**
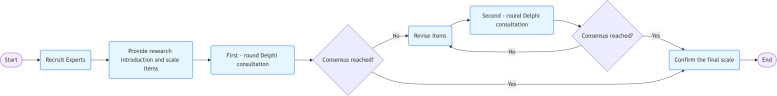
Delphi consensus process flowchart.

The PFLIE scale was developed and validated using a structured approach consisting of three phases. Item development: Domains and items were identified through a comprehensive literature review, clinical experience and cognitive interviews. Items were generated based on the Functional Living Index (FLIE) framework and adapted for pediatric cancer patients aged 8–18 years. The scale underwent two rounds of formal revision: Round 1: Experts rated the relevance of the items (5-point Likert scale) and provided feedback. Items related to nutritional status and physical fatigue were removed due to low correlation (I-CVI < 0.762). Round 2: Revised items were re-scored. Consensus (≥80% agreement) was reached and the final 20-item scale (10 nausea, 10 vomiting) was confirmed”. Scale development: Pre-testing included questioning of 15 patients, sampling and survey administration, item reduction and factor extraction. Cognitive interviews and pilot testing ensured clarity and feasibility of the scale. Scale evaluation: The dimensionality, reliability, and validity of the scale were tested through a formal survey of 90 pediatric cancer patients. Construct validity was assessed by Spearman’s correlations, concurrent validity by Ped-PRO-CTCAE comparisons, and internal consistency by Cronbach’s alpha (**Figure 1**).

### 2.2 Phase 1: scale development

This study primarily referred to the modified version of the FLIE with a 5-day recall (13), which has been widely used in adult patients. The development process involved cultural adaptation, literature review, clinical experience, and cognitive interviews, ultimately resulting in the PFLIE. Lindley et al. (12) and Martin et al. (13) previously described the development of the FLIE, which is a validated PROM specifically designed to assess the presence of nausea and vomiting and their impact on QoL. Since the original FLIE instrument was designed for adult cancer patients, it was necessary to adapt and modify items to be suitable for pediatric cancer patients aged 8–18 years. Such adjustments include rewording or modifying items to ensure that they are age-appropriate and understandable for children aged 8–18 years. This age group of pediatric cancer patients was specifically included in the scale because they are at a unique developmental stage with widely varying cognitive, language, and motor skills. Including this age group ensures that the scale reflects the diverse needs of pediatric patients while remaining clinically relevant. For example, the item “Has nausea (or vomiting) affected your daily functioning in the past 5 days?” was modified to “How much has nausea (or vomiting) affected your daily functioning in the past 5 days? (e.g., being able to eat, dress, use the bathroom, brush teeth, and wash face without assistance).” In addition, through qualitative interviews and the reporting of clinical care experiences of pediatric cancer patients (17), four items were added to each of the nausea and vomiting dimensions: “How much has nausea (or vomiting) affected your sleep in the past 5 days?,” “How much has nausea (or vomiting) affected your mood in the past 5 days?,” “How much has nausea (or vomiting) affected your nutritional status in the past 5 days?,” and “How much has your nausea (or vomiting) caused physical fatigue (e.g., lack of strength, feeling tired, not wanting to move) in the past 5 days?” Therefore, the PFLIE included 26 items, with 13 items categorized under the Nausea domain and 13 items under the Vomiting domain. It was observed that most children had difficulty understanding the concrete meaning of the intermediate scores on a 7-point scale and tended to select either the extreme values (1–3 points or 7 points). The decision to shorten the scale to a 4-point Likert scale was based on the results of a pilot test conducted with 15 pediatric cancer patients. During the course of the pilot test, participants reported difficulty distinguishing between the middle scores on the 7-point scale and tended to select the extremes (1–3 or 7). To address this issue, we modified the response options to a 4-point scale (1 = “not at all”, 2 = “slightly”, 3 = “generally”, 4 = “very much”). This modification improved clarity and consistency of responses, which was confirmed in the pilot survey where participants had a clear understanding of the wording and phrasing of each item.

To ensure objectivity, none of the 21 experts who assessed the content validity of the scale were involved in the initial development of the domain or program. The expert panel consisted of specialists in nursing administration, nursing psychology, nursing education, clinical oncology nursing, and child health care, recruited through purposeful sampling. Their credentials and professional backgrounds were carefully selected to provide different perspectives on the relevance and applicability of the scale.

The Delphi technique was employed to further refine the items and dimensions (18). A purposive sampling method was used to identify and recruit 21 Chinese experts, including specialists in nursing management, nursing psychology, nursing education, clinical oncology nursing, and child health care. The experts’ qualifications included: 2 (9.5%) with an associate degree, 14 (66.7%) with a bachelor’s degree, 4 (19.0%) with a master’s degree, and 1 (4.8%) with a doctoral degree. Their professional titles comprised 9 (42.9%) with intermediate titles, 5 (23.8%) with associate senior titles, and 7 (33.3%) with senior titles. The majority (71.4%) had more than 20 years of work experience in pediatric health care. The experts were from various regions across China, including South China (8, 38.1%), Central China (2, 9.5%), East China (5, 23.8%), North China (3, 14.3%), Southwest China (2, 9.5%), and Northeast China (1, 4.8%).

From August to October 2021, experts were provided with a consultation questionnaire that included a brief introduction to the research topic and the current dimensions and items of the PFLIE. Experts were asked to rate the importance of each dimension and item on a 5-point Likert scale, ranging from “not important” (1 point) to “very important” (5 points). To minimize potential differences in interpretation, the rationale for the revision of each item was provided in each round. Experts were also invited to provide comments on the content of the items and the survey as a whole, suggesting the addition or deletion of certain items and proposing any other necessary modifications, particularly regarding verbal expressions. The consultation questionnaires were sent to the experts via email, and they were requested to provide feedback within two weeks. The criteria for item retention and deletion were as follows: Item Retention: Items with an average importance score ≥3.5, a coefficient of variation ≤0.25, and a full score rate ≥20% were retained. Item Deletion: Items that did not meet all three criteria were deleted. Item Revision: If an item met two of the three criteria, it was revised based on expert suggestions. If an item met only one criterion, the research team decided whether to retain it, considering its importance and necessity in combination with clinical practice. After the first round, the revised scale was sent back to the experts for a second round of consultation.

In the first round, items related to nutritional status and physical fatigue were deleted. Considering that young pediatric patients may not be able to accurately report the impact of their nausea or vomiting symptoms on those closest to them, and parents typically do not display excessive worry or difficulty in front of their children, it was also recommended to delete the item on “hardship on others.” This is because the individuals who accompany patients to medical appointments, participate in consultations and therapy, or provide care may not be those closest to the patient, making it difficult for the patient to respond to this item. Consequently, the scale was adjusted to include 10 items in the Nausea domain and 10 items in the Vomiting domain, and the clarity of language expression was further improved. In the second round, the experts’ opinions tended to be consistent, and the consultation was discontinued. At this point, the final PFLIE, consisting of 20 items (10 items in the Nausea domain and 10 items in the Vomiting domain), was established (See **Supplementary Doc. S1**).

The Delphi technique is a widely used method for developing and validating research instruments. It facilitates the iterative collection of expert opinions to reach consensus on the content and structure of a scale. In this study, the Delphi method was used to refine the items and dimensions of the PFLIE to ensure its content validity and relevance to the target population. Experts were first recruited and then provided with a study brief and scale items. Then, two rounds of Delphi counseling were conducted. In each round, experts rated the importance of each item on a 5-point Likert scale. At the end of the first round, items were revised based on expert feedback, and the second round aimed to reach consensus. Finally, the final scale was confirmed (**Figure 1**).

### 2.3 Phase 2: pilot survey

The trial phase included 15 pediatric cancer patients (mean age: 12.3 ± 2.5 years; 53% male) with different types of cancer (53% leukemia, 27% lymphoma, 20% solid tumors). The characteristics of the sample corresponded to the target population (8–18 years old). The patients came from the oncology department of a tertiary hospital in Guangdong province. Subjects completed the PFLIE via a paper-and-pencil survey administered by trained interviewers during routine outpatient visits. Demographic and clinical data (e.g., cancer type, chemotherapy cycle, etc.) were also collected to ensure representativeness. Researchers conducted individual interviews to assess whether the participants had any difficulty understanding the scale and to examine their interpretations of all items. At the end of the test, the level of understanding was assessed through a semi-structured interview. All 15 pilot participants were able to understand the items correctly, with 100% reporting no ambiguity in wording. For example, when asked about “daily functions,” children referred to specific tasks (e.g., eating, dressing) without confusion. No modifications were needed based on feedback. The average time taken to complete the scale was approximately 5 minutes.

### 2.4 Phase 3: scale validation

The formal investigation was conducted at a tertiary care hospital in Guangdong Province, China, where 90 pediatric cancer patients were recruited between November 2021 and July 2022 from the cancer center. The sample size was precalculated using G* power (effect size = 0.3, α = 0.05, power = 0.90) and required a minimum of 84 participants. Taking into account possible dropouts, we recruited 90 patients. Inclusion criteria: 8–18 years old, diagnosed with cancer by pathologic evaluation (histologically confirmed), currently receiving chemotherapy, and written informed consent from parents. Exclusion criteria: patients with cognitive or psychiatric disorders, and patients who had not previously adhered to antiemetic medications. To ensure generalizability of the study, no exclusions were made based on tumor type. – This study was approved by the Institutional Review Board of Sun Yat-Sen University Cancer Center (approval number: B2021-113-01) and was conducted in accordance with the Declaration of Helsinki.

The formal survey was administered as follows Who? Face-to-face interviews by trained interviewers (nurses with experience in pediatric oncology). When? First day of chemotherapy (baseline) and day 6 of the cycle. How? Paper-and-pencil surveys were used; electronic tablets were trialed but deemed unnecessary because of the low literacy rate in this age group. Standardized: Interviewers received 2 hours of training on survey protocols, including standardized scripts and dealing with non-response questions. Inter-interviewer reliability was checked (κ = 0.89). Parents/guardians were present but did not provide assistance unless requested.

Several types of baseline demographic data, including age and sex, were collected upon hospital admission. All enrolled pediatric cancer patients completed the PFLIE scale on the first day of chemotherapy for training purposes and again on the 6th day during their chemotherapy cycle. Given the lack of a widely accepted assessment standard, study participants also completed the Pediatric Module of the Patient-Reported Outcomes version of the Common Terminology Criteria for Adverse Events (Ped-PRO-CTCAE) on the 6th day of their chemotherapy cycle, in addition to the newly developed PFLIE (19, 20).

### 2.5 Content validity

Statistical analysis was conducted using IBM SPSS Statistics 25.0 (IBM Corp., Armonk, NY, USA). Continuous and categorical variables were described using mean (± standard deviation) values and counts (percentages), respectively. Statistical significance was defined as P <.05. Content validity was assessed by expert ratings on a 4-point Likert scale (1 = highly irrelevant, 2=irrelevant, 3=relevant, 4 = highly relevant). The item-level content validity index (I-CVI) was calculated as the proportion of experts who rated an item as a 3 or 4. The Scale-Level Content Validity Index-Average Unweighted (S-CVI/UA) was calculated as the number of relevant items divided by the total number of scale items. The S-CVI/Ave was calculated as the average of all I-CVI values. S-CVI/UA>0.8 or S-CVI/Ave>0.9 indicated good content validity. All items met these thresholds, confirming PFLIE’s strong representation of its intended construct (21, 22).

### 2.6 Construct validity

Construct validity was assessed using Spearman’s correlation to examine the relationships between individual items and domain scores. A correlation coefficient greater than 0.4 between an item and its domain score suggests adequate convergent validity. Additionally, if the correlations between items within the same domain are stronger than those across different domains, it indicates that the scale has good discriminant validity (23).

### 2.7 Reliability

In the development of the questionnaire, both test-retest reliability and internal consistency were evaluated to ensure the instrument’s reliability. Test-retest reliability, also known as reproducibility, is assessed by administering the instrument to patients with stable conditions at two different time points. Internal consistency, or scale reliability, measures the degree to which items within a domain assess the same underlying concept. Due to the fluctuations in the frequency and severity of CINV during the first cycle of chemotherapy, it was not feasible to assess reproducibility. Therefore, the internal consistency of the PFLIE was evaluated using Cronbach’s alpha (24, 25). Cronbach’s alpha values greater than 0.75 indicate excellent internal consistency, while values exceeding 0.95 may suggest redundancy (13).

## 3 Results

### 3.1 Participant characteristics

The demographic and clinical characteristics of the participants are presented in **Table 1**. A total of 90 patients consented to participate in this study. The mean age of the participants was 12.18 ± 2.40 years, and 58.89% were male. Additionally, 33.33% of the participants reported a history of motion sickness, and 72% had experienced nausea and vomiting during previous chemotherapy.

**Table 1:** Baseline patient demographics and clinical characteristics (N = 90).

Characteristics	N = 90
Female sex, N (%)	37 (41.11)
Male sex, N (%)	53 (58.89)
Age (years)	
Mean ± SD	12.18 ± 2.40
Range	8–17
Weight, kg (mean ± SD)	42.32 ± 15.41
Height, cm (mean ± SD)	149.52 ± 19.56
Motion sickness history, n (%)	30 (33.33)
History of nausea and vomiting with previous chemotherapy, n (%)	72 (80.00)
Chemotherapy cycle number (mean ± SD)	10.98 ± 10.09

SD, standard deviation.

### 3.2 Validity of the PFLIE

#### 3.2.1 Content validity

After the 21 experts completed their evaluations, the I-CVI scores for the PFLIE domains ranged from 0.762 to 1.000, indicating satisfactory content relevance (**Table 2**). Additionally, the S-CVI/UA scores for both the Nausea and Vomiting domains were 0.933, and the S-CVI/Ave scores for these domains were also 0.933, suggesting an excellent level of content validity.

**Table 2:** Expert ratings and calculation of CVIs.

PFLIE item	Number of experts who awarded 3–4 points	I-CVI
Nausea domain		
1. How much nausea have you had in the past 5 days?	19	0.905
2. How much has nausea affected your ability to maintain usual recreation/leisure activities in the past 5 days? (e.g., reading, drawing, playing games, watching TV, taking walks)	21	1.000
3. How much has nausea affected your ability to do minor household tasks in the past 5 days? (e.g., tidying up toys, folding clothes, making beds)	16	0.762
4. How much has nausea affected your ability to enjoy meals in the past 5 days? (e.g., loss of appetite or feeling nauseous at the sight of food)	21	1.000
5. How much has nausea affected your ability to enjoy fluid refreshment in the past 5 days? (e.g., don’t want to drink water, milk, juice)	21	1.000
6. How much has nausea affected your willingness to see and spend time with family and friends in the past 5 days?	17	0.810
7. How much has nausea affected your daily functioning in the past 5 days? (e.g., being able to eat, dress, use the bathroom, brush teeth, wash face without assistance)	21	1.000
8. How much has nausea imposed a hardship on you (personally) in the past 5 days?	21	1.000
9. (Additional item 1) How much has nausea affected your sleep in the past 5 days? (e.g., difficulty falling asleep, easily waking up, shortened sleep duration)	20	0.952
10. (Additional item 2) How much has nausea affected your mood in the past 5 days? (e.g., irritability, anger outbursts, feeling down)	19	0.905
Vomiting domain		
11. How much vomiting have you had in the past 5 days?	18	0.857
12. How much has vomiting affected your ability to maintain usual recreation/leisure activities in the past 5 days? (e.g., reading, drawing, playing games, watching TV, taking walks)	21	1.000
13. How much has vomiting affected your ability to do minor household tasks in the past 5 days? (e.g., tidying up toys, folding clothes, making beds)	16	0.762
14. How much has vomiting affected your ability to enjoy meals in the past 5 days? (e.g., loss of appetite or feeling nauseous at the sight of food)	21	1.000
15. How much has vomiting affected your ability to enjoy fluid refreshment in the past 5 days? (e.g., don’t want to drink water, milk, juice)	21	1.000
16. How much has vomiting affected your willingness to see and spend time with family and friends in the past 5 days?	20	0.952
17. How much has vomiting affected your daily functioning in the past 5 days? (e.g., being able to eat, dress, use the bathroom, brush teeth, wash face without assistance)	19	0.905
18. How much has vomiting imposed a hardship on you(personally) in the past 5 days?	20	0.952
19. (Additional item 1) How much has vomiting affected your sleep in the past 5 days? (e.g., difficulty falling asleep, easily waking up, shortened sleep duration)	20	0.952
20. (Additional item 2) How much has vomiting affected your mood in the past 5 days? (e.g., irritability, anger outbursts, feeling down)	20	0.952

PFLIE, Pediatrics Functional Living Index—Emesis.

#### 3.3.2 Construct validity

The results of the correlation analyses demonstrated strong correlations between the items and their respective domains, with all correlation coefficients exceeding 0.6 (**Table 3**). Acceptable construct validity was observed, with stronger item-domain correlations within domains (*r* = 0.689-0.879) compared to those across domains (*r* = 0.514-0.775).

**Table 3:** Item–domain correlations for the construct validity of PFLIE (N = 90).

PFLIE item	Correlation with domain	
	Nausea	Vomiting
Nausea domain		
1. How much nausea have you had in the past 5 days?	0.743^a^	0.595^a^
2. How much has nausea affected your ability to maintain usual recreation/leisure activities in the past 5 days? (e.g., reading, drawing, playing games, watching TV, taking walks)	0.798^a^	0.685^a^
3. How much has nausea affected your ability to do minor household tasks in the past 5 days? (e.g., tidying up toys, folding clothes, making beds)	0.777^a^	0.694^a^
4. How much has nausea affected your ability to enjoy meals in the past 5 days? (e.g., loss of appetite or feeling nauseous at the sight of food)	0.813^a^	0.612^a^
5. How much has nausea affected your ability to enjoy fluid refreshment in the past 5 days? (e.g., don’t want to drink water, milk, juice)	0.852^a^	0.738^a^
6. How much has nausea affected your willingness to see and spend time with family and friends in the past 5 days?	0.775^a^	0.629^a^
7. How much has nausea affected your daily functioning in the past 5 days? (e.g., being able to eat, dress, use the bathroom, brush teeth, wash face without assistance)	0.691^a^	0.640^a^
8. How much has nausea imposed a hardship on you (personally) in the past 5 days?	0.859^a^	0.728^a^
9. (Additional item 1) How much has nausea affected your sleep in the past 5 days? (e.g., difficulty falling asleep, easily waking up, shortened sleep duration)	0.798^a^	0.700^a^
10. (Additional item 2) How much has nausea affected your mood in the past 5 days? (e.g., irritability, anger outbursts, feeling down)	0.765^a^	0.569^a^
Vomiting domain		
11. How much vomiting have you had in the past 5 days?	0.514^a^	0.689^a^
12. How much has vomiting affected your ability to maintain usual recreation/leisure activities in the past 5 days? (e.g., reading, drawing, playing games, watching TV, taking walks)	0.734^a^	0.832^a^
13. How much has vomiting affected your ability to do minor household tasks in the past 5 days? (e.g., tidying up toys, folding clothes, making beds)	0.694^a^	0.781^a^
14. How much has vomiting affected your ability to enjoy meals in the past 5 days? (e.g., loss of appetite or feeling nauseous at the sight of food)	0.729^a^	0.867^a^
15. How much has vomiting affected your ability to enjoy fluid refreshment in the past 5 days? (e.g., don’t want to drink water, milk, juice)	0.767^a^	0.861^a^
16. How much has vomiting affected your willingness to see and spend time with family and friends in the past 5 days?	0.724^a^	0.773^a^
17. How much has vomiting affected your daily functioning in the past 5 days? (e.g., being able to eat, dress, use the bathroom, brush teeth, wash face without assistance)	0.718^a^	0.817^a^
18. How much has vomiting imposed a hardship on you(personally) in the past 5 days?	0.775^a^	0.879^a^
19. (Additional item 1) How much has vomiting affected your sleep in the past 5 days? (e.g., difficulty falling asleep, easily waking up, shortened sleep duration)	0.712^a^	0.807^a^
20. (Additional item 2) How much has vomiting affected your mood in the past 5 days? (e.g., irritability, anger outbursts, feeling down)	0.657^a^	0.761^a^

PFLIE, Pediatrics Functional Living Index—Emesis. ^a^ *P* <.001.

#### 3.3.3 Concurrent validity

The Spearman correlation coefficients between the subscales of the PFLIE and the subscales of the PRO-CTCAE Symptom Terms are presented in **Table 4**. Significant relationships were observed between the PFLIE domain scores and the Nausea and Vomiting items of the Ped-PRO-CTCAE Symptom Terms, with correlation coefficients ranging from 0.691 to 0.813 (P < 0.001).

**Table 4:** The concurrent validity of the PFLIE (N = 90).

PFLIE Domain	Ped-PRO-CTCAE Symptom Term: Nausea	Ped-PRO-CTCAE Symptom Term: Vomiting
Nausea	0.762^a^	0.691^a^
Vomiting	0.708^a^	0.813^a^

PFLIE, Pediatrics Functional Living Index—Emesis; Ped-PRO-CTCAE, Pediatric Module of the Patient-Reported Outcomes version Of The Common Terminology Criteria For Adverse Events; ^a^ *P* <.001.

### 3.3 Reliability of the PFLIE

The internal consistency of the PFLIE was assessed using Cronbach’s alpha. The overall Cronbach’s alpha value for the PFLIE was 0.964, while the Cronbach’s alpha values for the Nausea domain and the Vomiting domain were 0.928 and 0.943, respectively. These high values suggest that the PFLIE has excellent internal consistency.

### 3.4 Comparison with existing tools

To better understand the performance of the PFLIE, we compared its psychometric properties to existing assessment tools for CINV in pediatric cancer patients. This comparison helps to highlight the strengths and limitations of the PFLIE and informs its use in clinical practice. The PFLIE has high content validity and internal consistency, similar to or better than some existing instruments. The inclusion of sleep and mood items in the PFLIE makes it more comprehensive in assessing the impact of CINV on the daily lives of pediatric patients. However, further research is needed to validate these findings in larger and more diverse populations (**Table 5**).

**Table 5:** Psychometric comparison of PFLIE with existing tools.

Tool Name	Number of Domains	Number of Items	Content Validity (CVI)	Cronbach’s α	Applicable Age Range	Key Features
PFLIE	2	20	Nausea and Vomiting domains: 0.933	Overall: 0.964; Nausea domain: 0.928; Vomiting domain: 0.943	8–18 years old	Culturally adapted for Chinese pediatric patients; Includes sleep and mood items
FLIE (Adult Version)	2	18	–	0.79	≥18 years old	Original tool; 5 - day recall period
Pediatric Nausea Assessment Tool	1	10	0.85	0.82	5–12 years old	Focuses on nausea frequency; No vomiting domain

## 4 Discussion

Despite significant advances in antiemetic therapies, CINV remain among the most distressing symptoms for children undergoing chemotherapy. These symptoms negatively affect patients’ quality of life and can lead to poor adherence to treatment, sometimes resulting in treatment suspension (26, 27). A survey revealed that “managing CINV’s impact on patients’ quality of life is the greatest challenge for oncology nurses (28). Therefore, using validated measurement tools to assess CINV and its impact on quality of life is crucial. While the current literature provides several standardized and validated tools for self-reported nausea in pediatric populations, such as the Pediatric Nausea Assessment Tool and the Adapted Rhodes Indexes of Nausea and Vomiting for Pediatrics (29, 30), these instruments do not comprehensively assess the full impact of CINV on the daily lives of pediatric patients. Specifically, they fail to capture aspects such as daily functioning, appetite, and family life, and thus cannot fully demonstrate the broader impact of chemotherapy on these young patients. The FLIE scale is a published and validated tool for assessing the impact of nausea and vomiting on the ability of adult cancer patients to maintain their daily life activities (13). Based on the content and framework of the original FLIE scale, we developed and validated the PFLIE scale, specifically designed to assess the impact of CINV on the daily lives of pediatric cancer patients. Our findings demonstrated that the PFLIE has satisfactory validity and reliability among Chinese pediatric patients, indicating that the PFLIE can serve as a valuable instrument for clinical assessment.

Some researchers have suggested that an acceptable instrument with sufficient content validity should have a CVI of > 0.80 (22). In this study, the S-CVI/UA score of the PFLIE was 0.933, and the S-CVI/Ave score was also 0.933, indicating that the instrument is highly relevant to the target measurement content. These high scores suggest that the PFLIE is a valid and unambiguous tool for assessing the presence of nausea and vomiting and their impact on the quality of life in pediatric cancer patients. Construct validity refers to the degree of correlation between the theoretical scale structure conceived by the researcher and the scale structure established by the survey results (31). In this study, we found that the item-domain correlations were stronger within domains than across domains, indicating that the PFLIE has good construct validity. This suggests that the PFLIE is a suitable instrument for evaluating the influence of CINV on daily functioning in pediatric cancer patients and is worthy of application in clinical settings. Concurrent validity was assessed by calculating the correlations between the PFLIE domain scores and the Nausea and Vomiting items of the Ped-PRO-CTCAE Symptom Terms scores. The correlation coefficients between the PFLIE domain scores and the Nausea and Vomiting items of the Ped-PRO-CTCAE Symptom Terms were significantly high and satisfactory. According to these results, the Chinese PFLIE demonstrates good concurrent validity. The Cronbach’s alpha value for the PFLIE was 0.964, while the Cronbach’s alpha values for the Nausea subscale and the Vomiting subscale were 0.928 and 0.943, respectively. These values all exceeded the acceptable coefficient level of 0.75 (13). The PFLIE demonstrated satisfactory and sufficient reliability, as indicated by the excellent internal consistency. From the above results, the PFLIE is a favorable pediatric-applicable instrument that can serve as an acceptable and reliable patient-reported outcomes tool for assessing the impact of CINV on the daily lives of pediatric cancer patients. The PFLIE may also enhance the understanding of pediatric patient outcomes, adding value to the improvement of care quality in daily clinical practice. Additionally, it can assist in evaluating the effectiveness of antiemetic drugs.

The PFLIE was validated as a favorable instrument for assessing the impact of CINV on the quality of life among pediatric cancer patients. Increased knowledge about pediatric patients’ quality of life after chemotherapy could lead to better identification and management of CINV, thereby facilitating improved health-related outcomes. However, there were some limitations in this study. We enrolled a sample of pediatric cancer patients from a single hospital in southern China, which limits the generalizability of our findings. Future research should aim to validate these findings by applying the instrument to pediatric cancer patients from other provinces in China. Additionally, the PFLIE scale was completed on the 6th day of the chemotherapy cycle, a time when some pediatric cancer patients may still be undergoing other treatments in the hospital setting. This may affect the assessment of patients’ daily functions in specific scenarios, such as their ability to perform household tasks and their willingness to see and spend time with family and friends. Therefore, further investigations are necessary to assess the applicability of the PFLIE scale among pediatric patients in both home and hospital settings, as well as among patients from other countries.

To address concerns about potential bias, we recognize that the inclusion of Ped-PRO-CTCAE may introduce confounders. Future studies should consider alternative validation methods that do not rely on secondary measurements. However, despite the limitations of the Ped-PRO-CTCAE as the gold standard for nausea/vomiting assessment, the initial decision to include it was based on its proven use in pediatric oncology.

## 5 Limitation

Limitations of this study include Underrepresentation of patients with no prior history of nausea/vomiting: approximately 80% of participants had experienced nausea/vomiting during prior chemotherapy, which may limit generalizability to untreated patients. Limited sample size (n=90): Although sufficient for initial validation, a larger group is needed to confirm robustness. Future studies should include a larger number of patients and explore replication in different populations. To date, no replication studies have been conducted, which remains a key next step. Additionally, as the Ped-PRO-CTCAE is not the gold standard for nausea/vomiting in QoL, future studies should explore alternative comparative metrics or establish new benchmarks for pediatric oncology symptom assessment.

## 6 Conclusion

This study detailed the development process and validated several psychometric properties of the Chinese version of the PFLIE. The instrument was found to be easy to complete and understand. We verified that the PFLIE demonstrated satisfactory content validity, construct validity, and concurrent validity, as well as excellent internal consistency in the Chinese population. All the evidence gathered indicates that the PFLIE is a reliable and suitable tool for assessing the impact of CINV on the quality of life among Chinese pediatric cancer patients. The PFLIE scale will assist healthcare providers in better identifying patients’ CINV, assessing its impact on daily functioning and quality of life, and providing more support resources for CINV symptom management in Chinese pediatric cancer patients.

We offer the following actionable recommendations for applying the PFLIE model in LMICs and rural areas: Training Programs: Develop training programs for healthcare providers in LMICs to effectively use PFLIE in their clinical practice. Resource Adaptation: Adapt the PFLIE to the local linguistic and cultural context to ensure its applicability and acceptability. Integration into clinical workflows: Integrate PFLIE into existing clinical workflows to minimize additional burden on healthcare providers. Community Engagement: Work with local community and patient advocacy organizations to raise awareness of the importance of CINV management and gather feedback on the usability of the tool. These strategies will help ensure that PFLIE can be effectively implemented in resource-limited settings, thereby improving the management of CINV and enhancing the quality of life for pediatric cancer patients worldwide.

## Data Availability

The raw data supporting the conclusions of this article will be made available by the authors, without undue reservation.
