# Supplementary material for: Development and validation of the Pediatrics Functional Living Index—Emesis scale
Source: Front Oncol. 2025 Jun 24;15:1573996. doi: 10.3389/fonc.2025.1573996 (PMC12234535; doi:10.3389/fonc.2025.1573996)
Supplement: Supplementary file 1 [file Table1.docx]

**Appendix 1:** Pediatrics Functional Living Index—Emesis (PFLIE)

Please use the following scores to answer these questions:

“Not at all” = 1

“Slightly” = 2

“Generally” = 3

“Very much” = 4

| **Nausea domain** | | | | | |
| --- | --- | --- | --- | --- | --- |
| 1. How much nausea have you had in the past 5 days? | **1** | **2** | **3** | **4** | **□** |
| 2. How much has nausea affected your ability to maintain usual recreation/leisure activities in the past 5 days? (e.g., reading, drawing, playing games, watching TV, taking walks) | **1** | **2** | **3** | **4** | **□** |
| 3. How much has nausea affected your ability to do minor household tasks in the past 5 days? (e.g., tidying up toys, folding clothes, making beds) | **1** | **2** | **3** | **4** | **□** |
| 4. How much has nausea affected your ability to enjoy meals in the past 5 days? (e.g., loss of appetite or feeling nauseous at the sight of food) | **1** | **2** | **3** | **4** | **□** |
| 5. How much has nausea affected your ability to enjoy fluid refreshment in the past 5 days? (e.g., don't want to drink water, milk, juice) | **1** | **2** | **3** | **4** | **□** |
| 6. How much has nausea affected your willingness to see and spend time with family and friends in the past 5 days? | **1** | **2** | **3** | **4** | **□** |
| 7. How much has nausea affected your daily functioning in the past 5 days? (e.g., being able to eat, dress, use the bathroom, brush teeth, wash face without assistance) | **1** | **2** | **3** | **4** | **□** |
| 8. How much has nausea imposed a hardship on you (personally) in the past 5 days? | **1** | **2** | **3** | **4** | **□** |
| 9. (Additional item 1) How much has nausea affected your sleep in the past 5 days? (e.g., difficulty falling asleep, easily waking up, shortened sleep duration) | **1** | **2** | **3** | **4** | **□** |
| 10. (Additional item 2) How much has nausea affected your mood in the past 5 days? (e.g., irritability, anger outbursts, feeling down) | **1** | **2** | **3** | **4** | **□** |
| **Vomiting domain** | | | | | |
| 11. How much vomiting have you had in the past 5 days? | **1** | **2** | **3** | **4** | **□** |
| 12. How much has vomiting affected your ability to maintain usual recreation/leisure activities in the past 5 days? (e.g., reading, drawing, playing games, watching TV, taking walks) | **1** | **2** | **3** | **4** | **□** |
| 13. How much has vomiting affected your ability to do minor household tasks in the past 5 days? (e.g., tidying up toys, folding clothes, making beds) | **1** | **2** | **3** | **4** | **□** |
| 14. How much has vomiting affected your ability to enjoy meals in the past 5 days? (e.g., loss of appetite or feeling nauseous at the sight of food) | **1** | **2** | **3** | **4** | **□** |
| 15. How much has vomiting affected your ability to enjoy fluid refreshment in the past 5 days? (e.g., don't want to drink water, milk, juice) | **1** | **2** | **3** | **4** | **□** |
| 16. How much has vomiting affected your willingness to see and spend time with family and friends in the past 5 days? | **1** | **2** | **3** | **4** | **□** |
| 17. How much has vomiting affected your daily functioning in the past 5 days? (e.g., being able to eat, dress, use the bathroom, brush teeth, wash face without assistance) | **1** | **2** | **3** | **4** | **□** |
| 18. How much has vomiting imposed a hardship on you(personally) in the past 5 days? | **1** | **2** | **3** | **4** | **□** |
| 19. (Additional item 1) How much has vomiting affected your sleep in the past 5 days? (e.g., difficulty falling asleep, easily waking up, shortened sleep duration) | **1** | **2** | **3** | **4** | **□** |
| 20. (Additional item 2) How much has vomiting affected your mood in the past 5 days? (e.g., irritability, anger outbursts, feeling down) | **1** | **2** | **3** | **4** | **□** |
